# Treatment of Locally Advanced Merkel Cell Carcinoma—A Multi-Center Study

**DOI:** 10.3390/cancers14020422

**Published:** 2022-01-14

**Authors:** Monika Dudzisz-Sledz, Paweł Sobczuk, Katarzyna Kozak, Tomasz Switaj, Hanna Kosela-Paterczyk, Anna Malgorzata Czarnecka, Slawomir Falkowski, Paweł Rogala, Tadeusz Morysinski, Mateusz Jacek Spalek, Marcin Zdzienicki, Tomasz Goryn, Marcin Zietek, Bozena Cybulska-Stopa, Stanisław Klek, Grazyna Kaminska-Winciorek, Barbara Ziolkowska, Anna Szumera-Cieckiewicz, Piotr Rutkowski

**Affiliations:** 1 Department of Soft Tissue/Bone Sarcoma and Melanoma, Maria Sklodowska-Curie National Research Institute of Oncology, 02-781 Warsaw, Poland; pawel.sobczuk@pib-nio.pl (P.S.); katarzyna.kozak@pib-nio.pl (K.K.); tomasz.switaj@pib-nio.pl (T.S.); hanna.kosela-paterczyk@pib-nio.pl (H.K.-P.); am.czarnecka@pib-nio.pl (A.M.C.); slawomir.falkowski@pib-nio.pl (S.F.); pawel.rogala@pib-nio.pl (P.R.); tadeusz.morysinski@pib-nio.pl (T.M.); mateusz.spalek@pib-nio.pl (M.J.S.); marcin.zdzienicki@pib-nio.pl (M.Z.); tomasz.goryn@pib-nio.pl (T.G.); piotr.rutkowski@pib-nio.pl (P.R.); 2 Department of Experimental and Clinical Physiology, Laboratory of Centre for Preclinical Research, Medical University of Warsaw, 02-097 Warsaw, Poland; 3 Department of Experimental Pharmacology, Mossakowski Medical Research Centre, Polish Academy of Sciences, 02-106 Warsaw, Poland; 4 Department of Oncology, Wrocław Medical University, 53-413 Wrocław, Poland; zietek.marcin@dco.com.pl; 5 Department of Surgical Oncology, Wrocław Comprehensive Cancer Center, 53-413 Wrocław, Poland; 6 Department of Clinical Oncology, Maria Sklodowska-Curie National Research Institute of Oncology, Cracow Branch, 31-115 Cracow, Poland; bcybulskastopa@vp.pl; 7 Department of Surgical Oncology, Maria Sklodowska-Curie National Research Institute of Oncology, Cracow Branch, 31-115 Cracow, Poland; z5klek@cyf-kr.edu.pl; 8 The Department of Bone Marrow Transplantation and Onco-Hematology, Skin Cancer and Melanoma Team, Maria Sklodowska-Curie National Research Institute of Oncology, Gliwice Branch, 44-102 Gliwice, Poland; Grazyna.Kaminska-Winciorek@io.gliwice.pl; 9 II Clinic of Radiotherapy & Chemotherapy, Maria Sklodowska-Curie National Research Institute of Oncology, Gliwice Branch, 44-102 Gliwice, Poland; Barbara.Ziolkowska@io.gliwice.pl; 10 Maria Sklodowska-Curie National Research Institute of Oncology, Department of Pathology and Laboratory Diagnostics, 02-781 Warsaw, Poland; anna.szumera-cieckiewicz@pib-nio.pl; 11 Institute of Hematology and Transfusion Medicine, Diagnostic Hematology Department, 02-776 Warsaw, Poland

**Keywords:** Merkel cell carcinoma (MCC), sentinel lymph node biopsy, radiotherapy, prognostic factors

## Abstract

**Simple Summary:**

Merkel cell carcinoma (MCC) is a rare skin cancer with unfavorable outcomes. Surgery remains the standard of care in the treatment of locally advanced disease. Perioperative radiotherapy and chemotherapy can be considered in selected patients. Analyzing 161 patients with locally advanced MCC treated with curative intent, we found that over one-third of patients developed disease recurrence. The use of perioperative radiotherapy decreased the risk of disease recurrence by over 50%. The 5-year overall survival rate was 55%. Moreover, we identified male gender, age above 70, metastases in lymph nodes at diagnosis, and no sentinel lymph node biopsy as factors associated with shorter overall survival.

**Abstract:**

Merkel cell carcinoma (MCC) is a rare, aggressive skin cancer with a high risk of recurrence and poor prognosis. The treatment of locally advanced disease involves surgery and radiotherapy. To analyze real-life treatment patterns and clinical outcomes, we conducted a retrospective analysis of data from 161 MCC patients treated with curative intent in four oncological centers in Poland. The median age at diagnosis was 72 years (30–94); 49.7% were male. Lymph node (LN) involvement at diagnosis was found in 26.9% of patients. Sentinel lymph node biopsy (SLNB) was performed in 36.5% of patients (positive in 10.5%), and 51.9% of patients received perioperative treatment. The relapse rate was 38.3%. With the median follow-up of 2.3 years, the median disease-free survival (DFS) was not reached, and the 1-year rate was 65%. The negative independent risk factors for DFS were male gender, metastases in LN at diagnosis, no SLNB in patients without clinical nodal metastases, and no perioperative radiotherapy. The estimated median overall survival (OS) was 6.9 years (95% CI 4.64–9.15). The negative independent risk factors for OS were male gender, age above 70, metastases in LN at diagnosis, and no SLNB in patients without clinical nodal metastases. Our results confirm that the MCC treatment should be conducted in an experienced multidisciplinary team; however, the outcomes are still unsatisfactory.

## 1. Introduction

Merkel cell carcinoma (MCC) is a rare neuroendocrine skin cancer that occurs primarily in the elderly. During recent years, the incidence has risen, primarily due to the aging of the population, increased sun exposure, use of immunosuppressive drugs, greater awareness, and improved diagnosis of MCC. The incidence rate of MCC is estimated at 0.25–0.32 per 100,000 persons annually and is 1.5 times higher in men than in women [1]. The risk factors for MCC include sun exposure, immunosuppression, immunodeficiency disorders such as AIDS, hematological malignancies, and Merkel cell polyomavirus (MCPyV) infection. The mainstay of the diagnosis is proper pathology examination and appropriate disease staging, according to the eighth edition of the American Joint Committee on Cancer (AJCC) TNM (tumor-node-metastases) criteria [1,2].

MCC is an aggressive neoplasm with a high risk of recurrence and poor prognosis. Treatment of locally advanced disease involves surgery followed by radiation therapy. Radiotherapy may be an alternative to surgery in patients ineligible for surgery due to comorbidities or poor performance status. Approximately 50–65% of MCC patients have localized disease at presentation, and 25–50% have regional metastases [3,4]. Subclinical nodal metastases are present in 30–50% of patients with primary MCC, with an increased presence in patients with a primary tumor greater than 1 cm in diameter [5,6,7,8,9,10,11,12,13,14]. The sentinel lymph node biopsy (SLNB) is recommended in cases without clinical nodal involvement. In patients with metastases to regional lymph nodes (stage III), therapeutic lymph node dissection (LND) is indicated [2]. Adjuvant radiotherapy following regional LND may improve treatment outcomes [15,16,17].

The median 5- years survival rate in the whole population of MCC patients ranges from 41% to 77% [4,5,18,19,20,21,22,23,24]. The incidence of locoregional and distant relapse is high, with a rate from 25% to 50% [18,19,20,21,22]. The factors associated with negative outcomes include HIV infection, chronic lymphocytic leukemia, T-cell immunosuppression, solid organ transplantation, primary tumor size above 2 cm, MCC of the head and neck, and lymphovascular invasion [1,2,18]. Nodal involvement is also related to worse outcomes [5,23]. Nearly 10% of MCC patients have distant metastases at diagnosis, and more than 30% of patients develop metastases during the course of the disease [24,25]. The prognosis in the unresectable and metastatic setting is unfavorable [5,19,23]. Treatment should be started promptly and carried out by an experienced multidisciplinary team [2]. Our study aimed to analyze the treatment outcomes of patients with locally advanced MCC (laMCC) treated in routine clinical practice in four reference oncology centers. The results show that male gender, nodal involvement at diagnosis, and no SLNB in patients without clinical metastases in LN are associated with poor prognosis in terms of disease-free survival (DFS) and overall survival (OS). Perioperative (neoadjuvant or adjuvant) radiotherapy improves the treatment outcomes and reduces disease progression risk but does not impact OS, while perioperative chemotherapy does not improve survival.

## 2. Materials and Methods

### 2.1. Patients Selection and Data Collection

This retrospective analysis included patients diagnosed with MCC who started treatment for locoregional diseases between 1 January 2010 and 31 December 2019. Patients were treated in four cancer centers in Poland experienced in MCC treatment: Maria Sklodowska-Curie National Research Institute of Oncology in Warsaw, Maria Sklodowska-Curie National Research Institute of Oncology—Gliwice Branch, Maria Sklodowska-Curie National Research Institute of Oncology—Cracow Branch, and Wrocław Comprehensive Cancer Center.

Medical records of all consecutive patients were screened. All patients with locally advanced MCC, localized disease (only primary tumor) or with metastases in lymph nodes but without distant metastases, treated with curative intent, were included in the study. All eligible patients had the diagnosis confirmed by pathologists experienced in skin cancer pathology. Patients with no information about front-line treatment, no confirmed diagnosis, or incomplete data were excluded from the analysis.

Collected data included the patients’ demographic data (gender, age at the diagnosis) and tumor-related information (tumor size, tumor location, UV exposure, type of diagnosis, lymph-nodes involvement, and metastatic sites). We also retrieved data concerning treatment modalities used with curative intent, e.g., dates and extend of surgical treatment (primary tumor resection, SLNB, LND), the number of resected lymph nodes, number of lymph nodes with tumor cells, and data on surgical margins. The surgical margins were coded as R0 (complete resection), R1 (microscopic residual tumor), or R2 (macroscopic residual tumor). Dates of locoregional or distant recurrence, death, or last follow-up were also retrieved.

### 2.2. Statistical Analyses

Patients were followed for survival status and disease recurrence. Local recurrence-free survival (LRFS), distant metastases-free survival (DMFS), and disease-free survival (DFS) were calculated from the date of radical treatment to the date of local recurrence, diagnoses of distant metastases, or any evidence of disease relapse whichever occurred first, respectively. Patients without signs of disease were censored at the last follow-up visit. Overall survival (OS) was calculated from the date of disease diagnosis up to death or last follow-up. Disease-specific survival (DSS) was calculated from the date of disease diagnosis up to the death due to disease. The patients alive at the date of data retrieval were censored (31 December 2020). Data were censored on 31 December 2020.

Descriptive statistics were used to report patients’ characteristics. DFS, OS, LRFS, and DMFS were calculated using the Kaplan–Meier method, and a log-rank test was used for assessing differences between survival curves. The Cox proportional hazard model was used to perform multivariable analysis, and all variables with *p*-value < 0.1 in univariate analysis were included. With point estimates, 95% confidence intervals (CI) were reported. All analyses and figure drawings were performed using IBM SPSS Statistics for Windows version 26 (IBM Corp., Armonk, NY, USA). The differences were considered statistically significant if the *p*-values were <0.05.

## 3. Results

### 3.1. Patients

**Figure 1:**
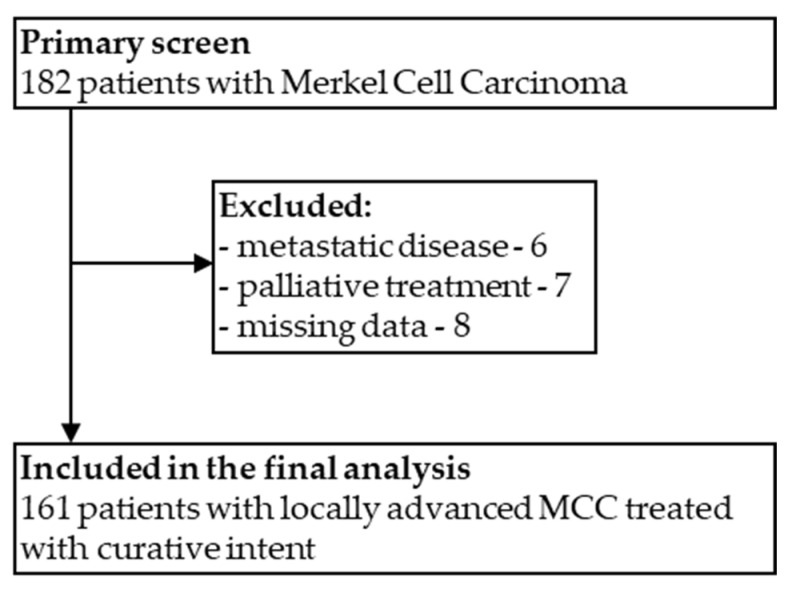
CONSORT flow diagram for inclusion and exclusion of cases in the study.

This retrospective analysis included 161 patients with MCC treated with curative intent (Figure 1). The median age was 72 (30–94), 55.9% of patients were above 70 years old, and 49.7% were male. Approximately 77.6% of patients had other comorbidities, with hypertension as the most prevalent. The most common primary tumor locations were lower limbs (32.9%), upper limbs (29.2%), and head and neck (29.2%). The primary origin was unknown in 4.3% of patients (MCCUP). In 59.6%, the primary tumor was located in the sun-exposed skin. The median tumor size was 25.5 mm (range 4–170). Clinical LN involvement at diagnosis was found in 28.6% of patients (*n* = 46). The detailed study population characteristics are summarized in Table 1.

### 3.2. Curative Treatment

One hundred sixty-one patients were treated, of whom 96.9% underwent surgery with or without perioperative treatment, while 3.1% received radiotherapy alone. Of 154 patients with the known primary origin, 150 (97.4%) underwent primary tumor resection. In 70.1% of patients, resections were performed outside participating reference centers. Negative surgical margins (R0) were achieved in 55.2% (85), while R1, R2, and unknown in 27.9% (43), 0.6% (1) and 13.6% (21), respectively. R0 resections were more often performed in the reference center than outside—85.7% vs. 45.4% (*p* < 0.001). Scar resection was performed in 51.3% (77) of patients. Tumor cells were found in the scar in 16.9% (13) of patients.

Sentinel lymph node biopsy (SLNB) was performed in 38% (57/150) of patients after primary tumor resection and was positive in 10.5% (6) of cases. In total, 26.7% (43) of patients treated with curative intent underwent lymph node dissection (LND), which appeared to be positive in 83.7% (36). All patients with positive SLNB had a completion LND performed with further nodal involvement found beyond SLN in 33.3% (2) of cases. An additional 12 patients underwent LND after completing curative treatment due to suspicion of locoregional recurrence—in this group, tumor cells were found in 66.7% (8) cases.

Perioperative treatment was administered in 81 of 156 patients who underwent curative surgery (51.9%). Of them, 21% (17) received chemotherapy and 86.4% (70) radiotherapy.

Fifteen patients received neoadjuvant chemotherapy with the median number of four cycles (range 1–6), and CAV (cyclophosphamide, doxorubicin, and vincristine)/PE (platinum and etoposide) was the most common regimen in 10 patients, followed by CAV or PE, each in 2 patients. Eight patients were treated with adjuvant chemotherapy, most commonly with CAV/PE regimen in seven cases, and the median number of four cycles (range 2–5).

Radiotherapy was mostly used postoperatively in 70.0% (49) of patients. Preoperative radiotherapy was more often used in patients without involvement of lymph nodes—71.4% (15/21) and in patients with larger tumor sizes—median 55 mm (range 22–170). The median dose of preoperative radiotherapy was 25 Gy (range 20–25) and 60 Gy for postoperative (range 20–70).

After completion of treatment, patients underwent routine follow-up every 3–6 months for the first 2–3 years and every 6–12 months thereafter. Follow-up visits included a physical exam (complete skin and node examination) and imaging (ultrasound, computed tomography) as clinically indicated.

### 3.3. Treatment Outcomes

**Figure 2:**
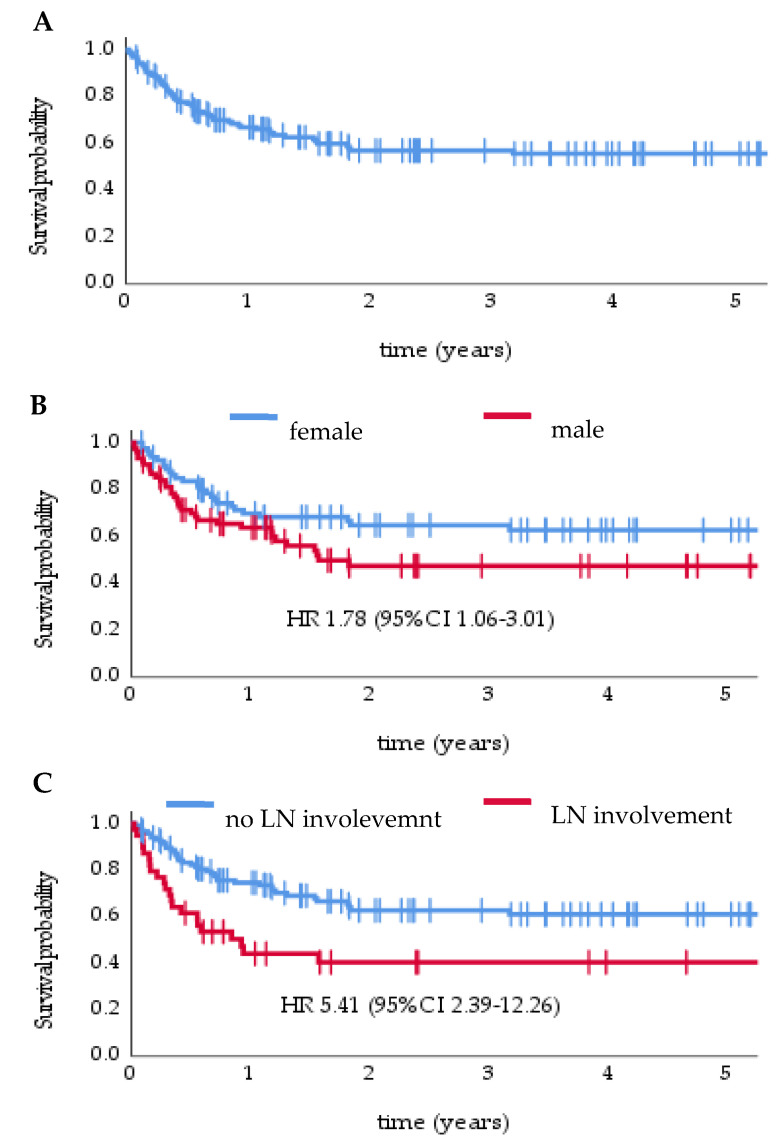
Disease-free survival in overall study population (**A**), stratified by gender (**B**) and lymph nodes (LN) involvement (**C**).

With a median follow-up of 2.3 (95% CI 1.97–2.64) years, disease recurrence was found in 40.4% (65) of patients—locoregional recurrence in 36.6% (59) and distant metastases in 12.4% (20). Median DFS was not reached, while the 1-, 2-, and 5-year DFS rates were 66%, 57%, and 55%, respectively (Figure 2A). Median local recurrence-free survival (LRFS) and distant recurrence-free survival (DMFS) were not reached. The 1-, 2-, and 5-year LRFS were 69%, 61%, and 59%, respectively, while DMFS were 92%, 86%, and 86%.

In a univariate analysis, male gender, lymph node involvement, no SLNB in patients without clinical nodal metastases, perioperative chemotherapy, and no perioperative radiotherapy were significant negative prognostic factors for DFS. In a multivariate Cox regression model, significant negative factors included: male gender (HR 1.42, 95% CI 1.06–3.01) (Figure 2B), lymph node involvement (HR 5.41, 95% CI 2.39–12.26) (Figure 2C), no SLNB in patients without clinical nodal metastases (HR 5.45, 95% CI 2.41–12.3) and no perioperative radiotherapy (HR 2.19, 95% CI 1.29–3.75) (Table 2).

**Figure 3:**
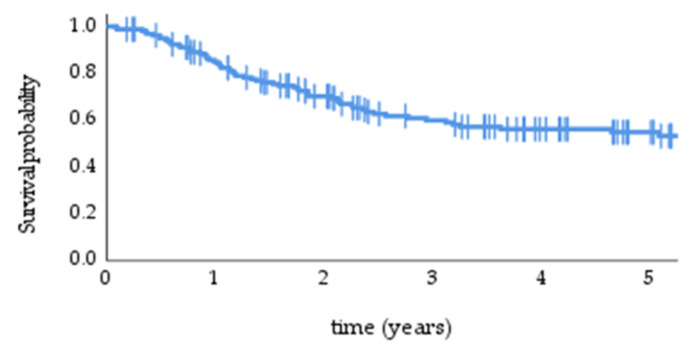
Overall survival in the study population.

During the follow-up, 42.2% (68) of the patients died—48.5% (33) patients died due to disease, and 51.5% (35) due to other causes. Median OS was 6.8 years (95% CI 3.56–10.03), with 1-, 2-, and 5-year OS of 85%, 70%, and 55%, respectively (Figure 3).

**Figure 4:**
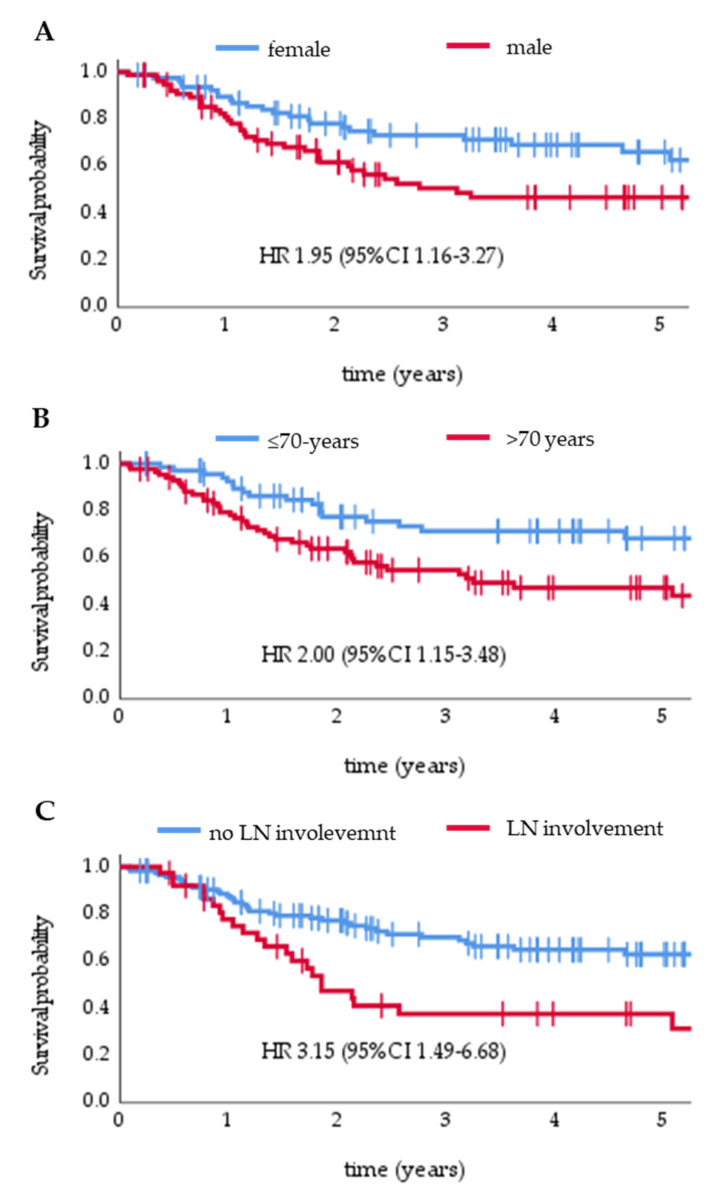
Overall survival is stratified by gender (**A**), age (**B**), and lymph nodes (LN) involvement at diagnosis (**C**).

In a univariate analysis, male gender, age ≥ 70 years, and lymph node involvement were significant negative prognostic factors for OS. In a multivariate Cox regression model, significant negative factors included: male gender (HR 1.95, 95% CI 1.16–3.27) (Figure 4A), age ≥ 70 years (HR 2.0, 95% CI 1.15–3.48) (Figure 4B), lymph node involvement (HR 3.15, 95% CI 1.49–6.68) (Figure 4C), and no SLNB in patients without clinical nodal metastases (HR 2.30, 95% CI 1.10–4.82) (Table 3).

Out of five patients treated with definitive radiotherapy, only one developed local recurrence after 6.6 months. Four patients died due to other causes, and the median OS was 30.1 months (95% CI 5.2–19.9). One patient is still alive and free of the disease for over seven years.

Median DSS was 8.6 years (95% CI 5.35–11.82), with 1-, 2-, and 5-year DSS rates of 94%, 88%, and 73%, respectively. In a univariate analysis, a lack of perioperative chemotherapy and lymph node involvement were significant negative prognostic factors for DSS, but in a multivariate model, only lymph node involvement (HR 3.15, 95% CI 1.49–6.68) was significantly associated with shorter DSS. The analysis is underpowered due to a low number of events and should be interpreted with caution.

## 4. Discussion

MCC is a rare and aggressive skin cancer with a high locoregional and distant recurrence rate. Due to the rarity of the disease, real-life evidence from routine clinical practice are of high importance to understand the natural course of the disease and improve care delivery. Here we present a large, national, multi-institutional analysis of patients with locally advanced MCC.

In the present study, the 5-year OS rate was 55%, which was consistent with available data indicating the rates ranging from 41% to 77% [5,19,20,23,24,26,27,28]. As previously reported, age ≥ 70 years was an independent negative prognostic factor. However, it is essential to highlight that MCC occurs primarily in elderly and frail patients; thus, a significant number of MCC patients die due to causes other than MCC [29]. Consistently, over 50% of deaths in our population were attributed to causes other than MCC. van Veenendaal et al. estimated that the 5-year risk of MCC-related death is 22% [29]. This finding corresponds to the 5-year DSS rate of 73% in our analysis.

Based on large meta-analyses, at least half of the patients with MCC develop locoregional relapse, and nearly one-third develop distant metastases [18,19,20,21,30]. In line with previous studies, we found that lymph node involvement was a significant negative prognostic factor for OS and DFS [5,23,29].

Based on the literature, occult lymph node disease is present in up to 50% of patients [5,6,7,8,9,10,11,12,13,14]; thus, SLNB should be performed in all patients. We confirmed the value of SLNB by showing that patients with clinically less-suspicious lymph nodes who have not undergone the SLNB had over 5-times higher risk of disease recurrence and nearly 2.5 times higher risk of death. These observations support evidence from previous studies. [31,32]. Furthermore, several reports suggest that negative SLNB is a predictor for improved outcomes [13,19,33,34].

Our study indicated that male gender is associated with a poor prognosis in terms of DFS and OS. Xia et al., in an analysis of data from 1973 patients with MCC, reported that female gender, among other factors (the primary site on the trunk, radiation, regional lymph nodes removed, SLNB, and SLNB +regional lymph nodes removed), is associated with better OS [32]. Another study also reported that male sex correlates with nodal involvement in patients with MCC [35]. There is no clear explanation for this phenomenon. One possible reason is a difference in immune responses between men and women [36]. Women have been shown to have more robust innate and adaptive immune responses than men and have approximately two to four times higher rates of systemic autoimmune diseases [37]. Moreover, improved outcomes in females have been described in numerous trials with immunotherapy [36]. More research analyzing the impact of gender on MCC prognosis is needed.

Due to the rarity of the disease, the diagnosis of MCC can be challenging. Current guidelines recommend performing a biopsy if skin cancer is suspected [2]; however, in our study, most patients were operated on without a prior biopsy. This could affect the high rate of R1 or R2 resections. Importantly, the R0 resection rate was significantly higher when patients were operated on in the experienced reference center, underlining the need to refer patients suspicious of MCC to dermatologists or surgeons experienced in this type of cancer.

Scar re-excision is not routinely performed if a wide surgical margin has been achieved. However, it should be considered in cases with positive or close margins after primary surgery or in high-risk patients (primary tumor > 2 cm in diameter, MCC of the head and neck, and immunocompromised patients) [2]. In our population, the rate of scar resection was higher than expected, most probably due to the low quality of primary surgery performed outside the reference center. In almost 20% of cases, MCC cells were found in the resected scar, justifying such an approach.

The role of perioperative treatment in MCC remains controversial. Previously, Chen et al. published an analysis of 4815 patients with MCC of head and neck, which indicated that adjuvant radiochemotherapy improves overall survival in high-risk patients [38]. The large retrospective analysis of 6908 patients with MCC revealed that adjuvant radiotherapy significantly improves OS in stage I and II MCC and does not improve OS in stage III MCC [17]. Some other studies suggest that radiotherapy improves only local control, with no effect on overall survival [29]. We have also found that adjuvant radiotherapy reduced the risk of disease recurrence by over 50% but did not affect overall survival. Despite the lack of evidence from randomized clinical trials, radiotherapy should be considered after surgery, especially in patients with narrow or positive surgical margins. Adjuvant radiotherapy after LND may also improve treatment outcomes [16,39].

There are no data to confirm the benefit of perioperative chemotherapy in patients with stage I-III MCC regarding OS [5,17,38,40,41]. A large meta-analysis of 52 trials showed similar recurrence rates for patients receiving chemo-radiotherapy or only radiotherapy in aperioperative setting [42]. Thus, chemotherapy is not routinely recommended but may be considered in some selected cases, such as marginally resectable lesions. Our analysis did not reveal any improvement in the treatment outcomes in patients who received perioperative chemotherapy.

Considering high MCC radiosensitivity, patients who are not suitable for surgery or refuse surgery may be offered definitive radiotherapy or radiochemotherapy [2]. Recent meta-analysis confirmed the noninferiority of this approach [42]. In our study, five patients treated with curative intent received radiotherapy alone, and only one disease recurrence after treatment was observed. The main limitation of our study is its retrospective character. Despite that, this study provides valuable data concerning the clinical management of MCC patients and underlines the importance of treatment in reference centers. Overall, the treatment outcomes for MCC patients are unsatisfactory, and the prognosis remains poor, especially in patients with nodal involvement. There is an unmet need for more effective treatment options for laMCC patients. New strategies, especially neoadjuvant immunotherapy, are currently tested in clinical trials, and the first results are promising [43]. Studies evaluating adjuvant strategies with immunotherapy or combined radiotherapy and immunotherapy are ongoing [44,45,46].

## 5. Conclusions

Based on the data analysis of patients with locally advanced MCC, male gender, nodal involvement at diagnosis, and no SLNB are associated with poor prognosis in terms of DFS and OS. Perioperative radiotherapy improves the treatment outcomes and reduces disease recurrence risk but does not impact OS. Perioperative chemotherapy does not improve survival. Our results are consistent with the results of previously published studies and confirm that treatment results of patients with laMCC are unsatisfactory and the prognosis is poor. Patients with MCC should be treated by an experienced multidisciplinary team in reference centers.

## Figures and Tables

**Table 1:** Demographic and clinical characteristics of the study population.

Factor		Patients % (*n*) *n* = 161
Gender	Male	49.7 (80)
Age	Median (range) [years]	72 (30–94)
	>70 years	55.9 (90)
Comorbidities *Hypertension* *Diabetes* *Coronary artery disease* *Arrhythmias* *Neoplasm* *Hypothyroidism*		77.6 (125) *50.9* (*82*) *23.0* (*37*) *21.7* (*35*) *12.4* (*20*) *8.7* (*14*) *6.8* (*11*)
Centre	Warsaw	59.6 (96)
	Cracow	15.5 (25)
	Gliwice	14.3 (23)
	Wrocław	10.6 (17)
Primary tumor size	Median (range) [mm]	25.5 (4–170)
	Missing data	35.4 (57)
Primary tumor location	Head and neck	29.2 (47)
	Trunk	4.3 (7)
	Upper extremities	29.2 (47)
	Lower Extremities	32.9 (53)
	MCCUP	4.3 (7)
UV-exposed skin *		59.6 (96)
Lymph nodes involvement at diagnosis		28.6 (46)
Distant metastases at diagnosis		0 (0)
Biopsy		28.0 (45)
Treatment	Surgery +/− perioperative (neoadjuvant/adjuvant) treatment	96.9 (156)
	Radiotherapy alone	3.1 (5)

Abbreviations: UV—ultraviolet, MCCUP—Merkel cell carcinoma of unknown primary origin, *—sites with prolonged exposition to UV, such as head and neck, distal parts of upper extremities or other areas based on the treating physician assessment.

**Table 2:** Univariate and multivariate analysis of factors associated with disease-free survival.

Factor		Univariate Analysis		Multivariate Analysis	
		Median DFS (95% CI) [Years]	*p*	HR (95% CI)	*p*
Gender	Female	NR	0.032	1	0.029
	Male	1.6 (0.8–2.3)		1.78 (1.06–3.01)	
Age	<70	NR	0.062	1	0.200
	70+	1.8 (0.1–3.7)		1.42 (0.83–2.41)	
Localisation of primary tumor	Head and neck	3.2 (NR-NR)	0.069	1	
	Trunk	NR		1.28 (0.26–6.18)	0.761
	Upper extremities	NR		0.83 (0.40–1.71)	0.611
	Lower Extremities	1.2 (0.4–1.9)		1.80 (0.88–3.71)	0.110
	MCCUP	NR		0.26 (0.03–2.20)	0.216
UV exposure	Yes	NR	0.779		
	No	NR			
Lymph nodes involvement	No	NR	<0.001	1	<0.001
	Yes	0.6 (0.0–1.2)		5.41 (2.39–12.26)	
SLNB performed in patients without clinical nodal metastases	Yes	NR	0.031	1	<0.001
	No	1.5 (0.7–2.4)		5.45 (2.41–12.30)	
Perioperative chemotherapy	Yes	0.6 (0.0–1.4)	0.002	1	0.170
	No	NR		0.62 (0.31–1.23)	
Perioperative radiotherapy	Yes	NR	0.031	1	0.004
	No	1.8 (NR-NR)		2.19 (1.28–3.75)	

Abbreviations: DFS—disease-free survival; HR—hazard ratio; NR—not reached; UV—ultraviolet; SLNB—sentinel lymph node biopsy; LN—lymph node; MCCUP—Merkel cell carcinoma of unknown primary origin.

**Table 3:** Univariate and multivariate analysis of factors associated with overall survival.

Factor		Univariate Analysis		Multivariate Analysis	
		Median OS (95% CI) [Years]	*p*	HR (95% CI)	*p*
Gender	Female	6.9 (5.8–8.0)	0.023	1	0.012
	Male	3.1 (0.8–5.4)		1.95 (1.16–3.27)	
Age	<70	NR	0.005	1	0.015
	70+	3.3 (0.7–5.8)		2.00 (1.15–3.48)	
Localisation of primary tumor	Head and neck	6.8 (4.1–9.5)	0.851		
	Trunk	NR			
	Upper extremities	NR			
	Lower Extremities	8.4 (1.2–15.5)			
	MCCUP	NR			
UV exposure	Yes	6.8 (3.2–10.4)	0.358		
	no	NR			
Lymph nodes involvement	no	10.1 (NR-NR)	0.001	1	0.003
	yes	1.9 (1.3–2.4)		3.15 (1.49–6.68)	
SLNB performed in patients without clinical nodal metastases	Yes	8.6 (5.2–12.0)	0.094	1	0.027
	no	4.6 (0.8–8.5)		2.30 (1.10–4.82)	
Perioperative chemotherapy	Yes	3.3 (0.0–7.5)	0.257		
	No	6.9 (4.6–9.2)			
Perioperative radiotherapy	Yes	NR	0.072	1	0.056
	No	4.6 (1.8–7.5)		1.67 (0.99–2.83)	

Abbreviations: DFS—disease-free survival; HR—hazard ratio; NR—not reached; UV—ultraviolet; SLNB—sentinel lymph node biopsy; LN—lymph node; MCCUP—Merkel Cell Carcinoma of unknown primary origin.

## Data Availability

All data generated or analyzed during this study are available upon reasonable request upon DTA agreement.
